# Impact of myocardial phenotype on optimal atrioventricular delay settings during biventricular and left bundle branch pacing at rest and during exercise: insights from a virtual patient study

**DOI:** 10.1093/europace/euaf082

**Published:** 2025-04-08

**Authors:** Claudia A Manetti, Nick van Osta, Ahmed S Beela, Lieven Herbots, Frits W Prinzen, Tammo Delhaas, Joost Lumens

**Affiliations:** Department of Biomedical Engineering, CARIM School for Cardiovascular Diseases, Maastricht University Medical Center, Universiteitssingel 40, 6229 ERMaastricht, The Netherlands; Department of Biomedical Engineering, CARIM School for Cardiovascular Diseases, Maastricht University Medical Center, Universiteitssingel 40, 6229 ERMaastricht, The Netherlands; Department of Biomedical Engineering, CARIM School for Cardiovascular Diseases, Maastricht University Medical Center, Universiteitssingel 40, 6229 ERMaastricht, The Netherlands; Department of Cardiovascular Diseases, Faculty of Medicine, Suez Canal University, Ismailia 41522, Egypt; Department of Cardiology, Hartcentrum Hasselt, Jessa Hospital, Hasselt, Belgium; Faculty of Medicine and Life Sciences, Biomedical Research Institute, Hasselt University, Hasselt, Belgium; Department of Physiology, Cardiovascular Research Institute Maastricht (CARIM), Maastricht University, Maastricht, The Netherlands; Department of Biomedical Engineering, CARIM School for Cardiovascular Diseases, Maastricht University Medical Center, Universiteitssingel 40, 6229 ERMaastricht, The Netherlands; Department of Biomedical Engineering, CARIM School for Cardiovascular Diseases, Maastricht University Medical Center, Universiteitssingel 40, 6229 ERMaastricht, The Netherlands

**Keywords:** Atrio-ventricular delay optimization, Computational modelling and simulation, CircAdapt, Stiffness, *In silico* trial

## Abstract

**Aims:**

Previous studies have not examined the role of non-electrical myocardial disease substrates in determining the optimal atrio-ventricular delay (AVD) settings. We conducted virtual patient simulations to evaluate whether myocardial disease substrates influence the acute response to AVD optimization at rest and during exercise.

**Methods and results:**

The CircAdapt cardiovascular model was used to simulate various left ventricular (LV) remodelling found in cardiac resynchronization therapy candidates. We simulated electrical dyssynchrony, LV dilatation with preserved and reduced contractility, and increased LV passive stiffness. We simulated cardiac resynchronization following biventricular (BiVP) and non-selective LBB pacing (nsLBBP). The paced-AVD ranged from 220 to 40 ms. Cardiac output and heart rate were increased to simulate different levels of exercise. The optimal AVD was the one leading to the highest stroke volume (SV) and the lowest mean left atrial pressure (mLAP). At rest, in simulations with healthy myocardium the gain in SV by AVD optimization was larger compared to those with reduced contractility and stiff myocardium. However, mLAP was comparably decreased by AVD optimization in both healthy and diseased myocardium. During exercise, the optimal AVD shifted to shorter values, and mLAP was more sensitive to AVD, particularly in the presence of hypo-contractile and stiff myocardium.

**Conclusion:**

Simulations show that hypocontractility and stiffness reduce the effect of AVD optimization on SV but enhance its benefit in lowering mLAP. Notably, virtual patients with stiff ventricles experience greater benefits from AVD optimization during exercise compared to resting conditions. Furthermore, nsLBBP provides more favourable improvements in mLAP than BiVP.

## Graphical Abstract

**Graphical Abstract:**
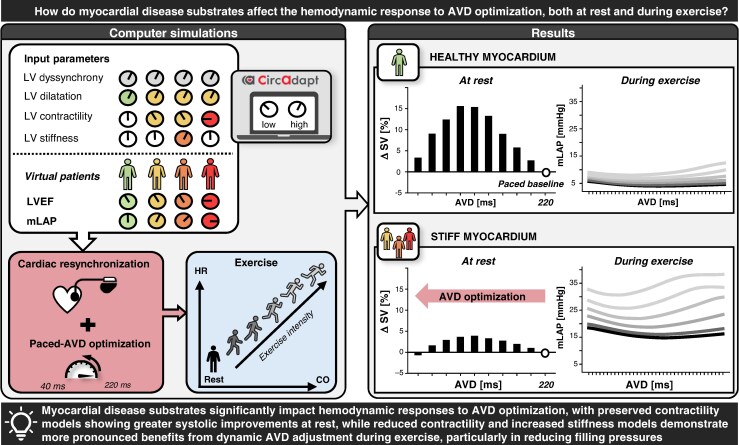

**What’s new?**

- The response to atrioventricular delay (AVD) optimization is modulated by a patient's left ventricular myocardial disease. Both reduced contractility and increased passive stiffness make left-heart filling pressure more sensitive to AVD, in particular under exercise conditions.
- The acute haemodynamic response to AVD optimization at rest, in terms of systolic blood pressure, maximum rate of left ventricular pressure change, and stroke volume, may not accurately reflect the potential haemodynamic benefits during exercise. The choice of the optimal AVD varies for each patient.
- In the presence of myocardial disease substrates that increase operational myocardial stiffness, optimal AVD decreases more steeply with increasing heart rate during exercise.

## Introduction

Cardiac resynchronization therapy (CRT) is a well-established and recommended treatment for patients with heart failure (HF), who have left bundle branch block (LBBB) and a reduced left ventricular ejection fraction (LVEF < 35%).^1^ CRT functions via two distinct physiological mechanisms. First, through (partial) resynchronization of ventricular activation, CRT augments systolic performance by increasing ventricular contractility and improving pump efficiency.^2–5^ Secondly, CRT co-ordinates the timing of atrial and ventricular contractions by shortening the pathologically prolonged atrio-ventricular delay (AVD). This shortening increases ventricular filling time without truncating the active filling wave, thereby enhancing left ventricular (LV) filling and improving diastolic function.^2,6^

Previous clinical studies have concentrated on establishing the appropriate methodology for determining the optimal AVD, either being sensed or paced.^6–11^ Notably, most proposed methodologies for optimization of pacing delays rely solely on data acquired during resting conditions. Nonetheless, optimizing AVD could be especially advantageous during exercise, as many HF patients experience symptoms during physical exertion. An inadequate increase in cardiac output (CO) during exertion or the uncomfortable sensation of dyspnoea caused by pulmonary venous congestion and increased respiratory muscle work due to elevated cardiac filling pressures can limit exercise and functional capacity.^12^ Therefore, mean left atrial pressure (mLAP), used as a measure of filling pressure, could potentially serve as an important marker.

Furthermore, few studies explored the degree to which underlying myocardial conditions influence the optimal settings among patients. Understanding and distinguishing the potential effects of these phenotypic factors on the response to AVD optimization during resting and exercise conditions is complex in a clinical environment. Therefore, computational models of the human cardiovascular system hold promise in providing valuable insights into the individual and combined impacts of each factor on treatment outcomes.^2,9,10,13^

We hypothesize that the potential effects of pacing-induced changes of AVD on cardiac pump function are modulated by non-electrical myocardial disease substrates, such as contractile dysfunction and myocardial stiffness, as well as by exercise-induced changes in cardiac loading condition. The aim of the present study is to provide mechanistic insight into how typical HF-related myocardial disease substrates impact the functional response to paced AVD optimization, both at rest and during exercise. Specifically, we examine two resynchronization strategies—biventricular pacing (BiVP) and non-selective LBB pacing (nsLBBP)^13^—which differ in their mechanisms of action and may lead to distinct responses in optimal AVD settings.

## Methods

The CircAdapt model of the human heart and circulation (www.circadapt.org)^14,15^ was used to simulate virtual pacing therapy and a related AVD optimization protocol in a set of virtual HF patients with distinct LV myocardial phenotypes, characterized by isolated or combined systolic and diastolic dysfunction. To study the effect of AVD optimization during exercise, we repeated the same virtual resynchronization and AVD optimization protocol at increasing levels of exercise intensity. The CircAdapt model enables fast and realistic beat-to-beat simulation of cardiovascular mechanics and haemodynamics under various (patho-) physiological conditions. Many previous studies have demonstrated the robustness of the CircAdapt modelling framework for simulating cardiovascular physiology during cardiac pacing,^16–19^ AVD optimization,^2,9,10^ and exercise.^20,21^

## Model description

**Figure 1:**
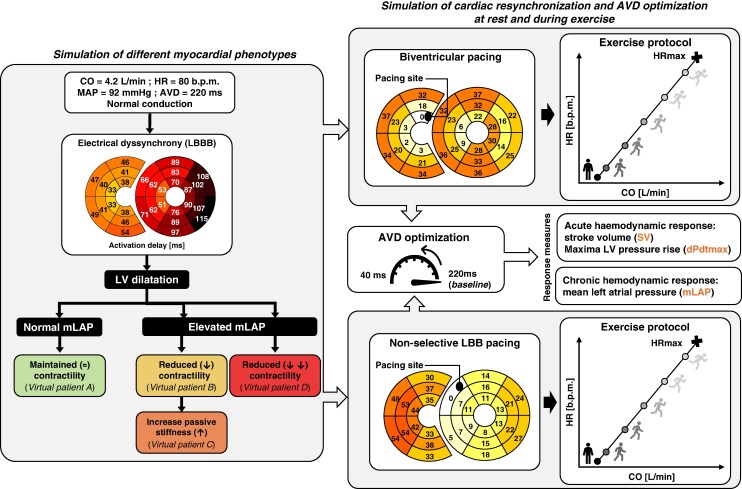
Overview of the simulation protocol used in this study. The first box shows the simulation of different HF phenotypes. Starting from the default reference with prolonged AVD, an LBBB-like ventricular activation pattern was imposed, followed by the simulation of dilatation (EF < 35%) with maintained, reduced contractility. We imposed an increase in myocardial stiffness until an mLAP exceeded 15 mmHg. The top and bottom boxes represent the simulation of cardiac pacing (i.e. BiVP and nsLBBP) and AVD optimization at rest and during exercise. The optimal paced AVD at rest was assessed using SV and mLAP which is used as a measure of LV filling pressure. AVD, atrio-ventricular delay, CO, cardiac output; EF, ejection fraction; HF, heart failure; HR, heart rate; LBBB, left bundle branch block; LV, left ventricular; mLAP, mean left atrial pressure; nsLBBP, non-selective LBB pacing; SBP, systolic blood pressure; SV, stroke volume.

CircAdapt is configured as a network of modules, each representing a functional component of the closed-loop cardiovascular system, including myocardial tissue, cardiac walls, cardiac valves, the pericardium, large blood vessels, and systemic and pulmonary peripheral vasculature. The ventricles consist of three myocardial walls,^14^ i.e. the LV free wall (LVfw), the interventricular septum (IVS), and the RV-free wall (RVfw). The mechanical interaction between these walls and the haemodynamic interaction through the systemic and pulmonary circulation ensures realistic interventricular interactions and closed-loop system physiology.^14^ The Multipatch module allows for subdivision of each cardiac wall into mechanically coupled segments, thereby enabling simulation of heterogeneous tissue properties in the cardiac walls.^15^ The LVfw and IVS were subdivided into 12 and 6 segments, in accordance with the 18 American Heart Association (AHA) segmentation.^22^ The RVfw was divided into 12 segments, resulting in a total number of 30 ventricular myocardial segments. The below sections and *Figure 1* explain the simulation protocol used in this study.

### Virtual patient simulations

The default implementation of the CircAdapt model represents a healthy virtual subject with normal values for CO (5.1 L/min), mean arterial pressure (MAP, 92 mmHg), heart rate (HR, 70 b.p.m.), AVD (150 ms), and with synchronous electromechanical activation of the ventricular walls. A virtual LBBB patient was simulated as described in,^16^ by setting CO to 4.2 L/min, HR to 80 b.p.m., prolonging AVD to 220 ms, and by imposing a typical LBBB-pattern of ventricular activation, obtained from an Eikonal model applied to a 3D biventricular mesh as described previously^18^ (*Figure 1*: *bullseye plot*). The experimental design incorporated an intentionally prolonged baseline AVD to reflect the pathophysiologically extended PR interval commonly observed in CRT candidates.^23–25^ This approach enabled systematic AVD shortening to evaluate its impact on cardiac performance while preventing fusion pacing.

Following the methodology established by Beela *et al.*^26^ we generated four distinct virtual patient simulations with reduced LVEF ( < 35%) derived from the baseline LBBB simulation. Each simulation represented a unique myocardial phenotype resulting in either normal or elevated filling pressures. The methodological approach largely paralleled that described in the aforementioned study, as detailed below:

#### Left ventricular dilatation with normal filling pressure

Left ventricular dilation was simulated by increasing both cardiac wall mass and wall area to 130% of their respective reference values. This adjustment ensured that the ratio between the total LV wall volume (i.e. the sum of LVfw volume and septal wall volume) and the cavity volume remained constant. The increase in wall area and mass resulted in an EF < 35% and end-diastolic volume (EDV) of 197 mL, consistent with EDV values typically observed in HF patients.^25^ At the tissue level, the operating sarcomere length, along with contractile function and passive mechanical properties, remained unchanged. As a result, the increase in LV EDV is achieved without elevating filling pressure [*Figure 1*: Virtual patient A, ‘Maintained (≈) Contractility’].

#### Left ventricular dilatation with elevated filling pressure

Two distinct approaches were employed to simulate elevated filling pressure:

- *Decreased contractility with increased passive stiffness:* LV dilatation was obtained by reducing contractility (i.e. isometric active myofiber stress) to 60% of reference values in the IVS and LVfw. This reduction was chosen to match the decreased maximum rate of LV pressure rise (dPdtmax) observed in HF patients.^25^ In this scenario, the sarcomere’s ability to generate force was reduced across all sarcomere lengths. As a result, the sarcomeres must operate at longer lengths to produce the necessary contractile force. At the organ level, this resulted in reduced LVEF and dPdtmax with concurrent increases in EDV and filling pressure [*Figure 1*: virtual patient B, ‘Reduced (↓) contractility’].
  Building on this scenario, we increased the passive stiffness exponent, scaling the material stiffness of extracellular structures of the IVS and LVfw, to 140% of their reference values. This adjustment simulated diastolic dysfunction by raising mLAP to 18 mmHg, exceeding the 15 mmHg clinical threshold distinguishing normal from elevated filling pressures.^27^ This scenario represents a more advanced stage of diastolic dysfunction and provides valuable insights into the impact of increased myocardial stiffness on cardiac performance [*Figure 1*: virtual patient C, ‘Increased Passive Stiffness (↑)’].
- *Severe contractility reduction:* To explore a scenario of further increased LV dilation with an elevated mLAP, we further reduced isometric active myofiber stress in the IVS and LVfw to 45% of the reference values in the baseline LBBB simulation. This reduction was chosen to simulate cases with a low EF of 20% and severely dilated LV with EDV of 252 mL^25^ [*Figure 1*: virtual patient D, ‘Reduced (↓↓) Contractility’].

A more detailed description of the methodologies used is provided in the Supplement of Beela *et al.*^26^

### Virtual pacing protocol

For all baseline virtual patient simulations, we applied BiVP and nsLBBP as simulated by Meiburg *et al.*^18^ (*Figure 1*). Cardiac resynchronization was followed by AVD optimization, as described below, with the RV activation pattern remaining unchanged across different AVD settings, as we assumed full ventricular capture.

The paced AVD was progressively reduced from 220 to 40 ms in 20 ms decrements. To evaluate both immediate beat-to-beat functional effects and longer-term haemodynamic consequences of AVD optimization, simulations were conducted under two distinct conditions: without and with homeostatic regulation of arterial blood pressure and systemic flow. The non-regulated simulations provide insight into very acute haemodynamic changes before compensatory mechanisms activate, while regulated simulations elucidated longer-term effects after regulation of preload and systemic vascular resistance, such that MAP and CO were maintained at their baseline values. In simulations without homeostatic regulation, MAP and CO varied according to simulation parameters, with stroke volume (SV) and dPdtmax serving as primary outcome variables. Stroke volume, derived from aortic flow patterns, represents a clinically accessible measure of LV performance that reflects the efficiency of both ventricular filling and ejection—critical components in evaluating CRT outcomes. Conversely, when homeostatic regulation was activated, MAP and CO were maintained at target values, while mLAP functioned as the primary outcome measure, varying in response to paced AVD adjustments. This dual methodological approach enabled comprehensive evaluation of AVD optimization effects across different physiological timeframes. Following Manisty *et al.*^28^ we also analysed systolic blood pressure (SBP) variations as a surrogate for SV, specifically examining SBP changes during the initial 10 cardiac cycles following AVD reduction from 120 to 40 ms (see Supplementary material online, *Figure S1*).

### Virtual exercise protocol

Cardiovascular exercise dynamics can be simulated using the CircAdapt model as described previously.^20,21^ In brief, HR and cardiac preload are increased such that a target CO is achieved. On average, HF patients need a larger increase of HR to achieve a target CO, as compared to control subjects.^29^

**Figure 2:**
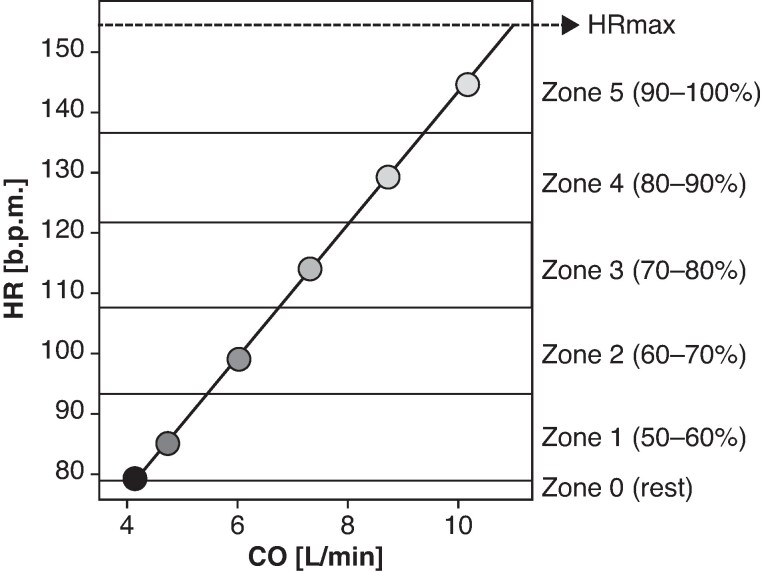
Exercise protocol divided into six zones, each calculated as a percentage of the maximum heart rate (HRmax), which is determined using the fox formula (HRmax = 220–age). The calculation is based on a virtual patient aged 65 years. CO, cardiac output.

To represent the typical profile of a CRT candidate, the haemodynamic of a 65-year-old virtual patient were modelled, reflecting the average age of CRT patients,^25,30,31^ with a maximum HR of 155 b.p.m., calculated using the Fox formula (220–age).^32^ Exercise intensity zones were defined as 50–60, 60–70, 70–80, 80–90, and 90–100% of maximum HR (*Figure 2*). Starting from a baseline HR of 80 b.p.m. and a CO of 4.2 L/min, the five exercise stages were established based on mid-range HR and CO values within each exercise zone, assuming a maximum CO of 11 L/min. *Table 1* presents the HR and CO values used in the exercise protocol, which align with those reported by Pugliese *et al.*^29^ for patients with HFrEF. At each exercise zone, the virtual pacing protocol with the different AVDs was applied, but now with steps of 5 ms instead of 20 ms. Assuming that exercise capacity is constrained by elevated filling pressure,^21,33^ we computed mLAP values for all AVD settings, while forcing CO to the target values of the exercise ones with homeostatic regulation as described above.

**Table 1:** Exercise protocol: this table presents HR, CO, and SV values corresponding to different exercise zones, starting from rest (zone 0) to maximum effort (HR max)

Zone	HR (b.p.m.)	CO (L/min)	SV (mL)
0 (Rest)	80	4.2	52.48
1	86.5	4.79	55.34
2	100.5	6.06	60.26
3	115.5	7.42	64.20
4	130.5	8.78	67.26
5	146	10.23	69.84
HR max	155	11	–

CO, cardiac output; HR, heart rate; SV, stroke volume.

## Results

*Table 2* shows LV haemodynamics for all four baseline LBBB simulations with reduced EF. Though LVEF was similar in all virtual patients with dilated LVs, filling pressure (i.e. mLAP) was much higher in the presence of reduced contractility and increased passive stiffness of the LV myocardium. dPdtmax was higher when contractility was maintained compared to the group of reduced contractility.

**Table 2:** Simulated baseline left ventricular haemodynamics of the four virtual patients with LBBB and reduced EF

	EF (%)	EDV (mL)	mLAP (mmHg)	dPdtmax (mmHg/s)
Virtual patient A	27	197.3	5.6	950.8
LBBB + LV dilatation with maintained contractility				
Virtual patient B	27	196.5	11.3	783.72
LBBB + LV dilatation with reduced contractility				
Virtual patient C	29	180.1	17.3	779.8
LBBB + LV dilatation with reduced contractility + Increase passive stiffness				
Virtual patient D	21	252.4	19.2	662.7
LBBB + LV dilatation with severely reduced contractility				

dPdtmax, maximum rate of LV pressure change; EDV, end-diastolic volume; EF, ejection fraction; LBBB, left bundle branch block; LV, left ventricular; mLAP, mean left atrial pressure.

### Effect of cardiac pacing on left ventricular haemodynamics

*Table 3* presents the baseline LV haemodynamic parameters for the four virtual patients under three conditions: Unpaced (LBBB, AVD = 220 ms), BiVP (AVD = 220 ms), and nsLBBP (AVD = 220 ms). Comparing BiVP and nsLBBP, the results show that both pacing modalities produce similar improvements in SV and dPdtmax, with only marginal differences. However, nsLBBP consistently achieves slightly higher dPdtmax values across all patients, indicating potentially more effective electromechanical resynchronization compared to BiVP. Regarding SV, the differences between BiVP and nsLBBP are minimal. Notably, nsLBBP appears to be more effective in reducing mLAP, with all virtual patients demonstrating lower mLAP values compared to BiVP. The differences are most evident in virtual patients A and B, where nsLBBP results in a greater reduction of mLAP compared to BiVP.

**Table 3:** Simulated haemodynamic data for the four virtual patients, comparing the unpaced state (LBBB, AVD = 220 ms) with the paced baseline (AVD = 220 ms) before AVD optimization.

	Unpaced (LBBB, AVD = 220 ms)	BiVP paced baseline (AVD = 220 ms)	nsLBBP paced baseline (AVD = 220 ms)
Virtual patient A			
SV (mL)	52.4	56.5	56.05
dPdtmax (mmHg/s)	954.5	1075.4	1078.0
mLAP (mmHg)	5.6	4.61	4.2
Virtual patient B			
SV (mL)	52.4	56.6	57.2
dPdtmax (mmHg/s)	787.7	885.2	908.4
mLAP (mmHg)	11.3	9.12	8.4
Virtual patient C			
SV (mL)	52.4	54.7	55.1
dPdtmax (mmHg/s)	781.3	873.1	886.1
mLAP (mmHg)	17.3	14.9	13.8
Virtual patient D			
SV (mL)	52.4	55.1	55.9
dPdtmax (mmHg/s)	665.7	733.1	742.0
mLAP (mmHg)	19.2	16.2	14.8

AVD, atrio-ventricular delay; dPdtmax, maximum rate of LV pressure change; LBBB, left bundle branch block; LV, left ventricular; mLAP, mean left atrial pressure; nsLBBP, non-selective LBB pacing; SV, stroke volume.

### Effect of atrio-ventricular delay variation and cardiac pacing on left ventricular haemodynamics

**Figure 3:**
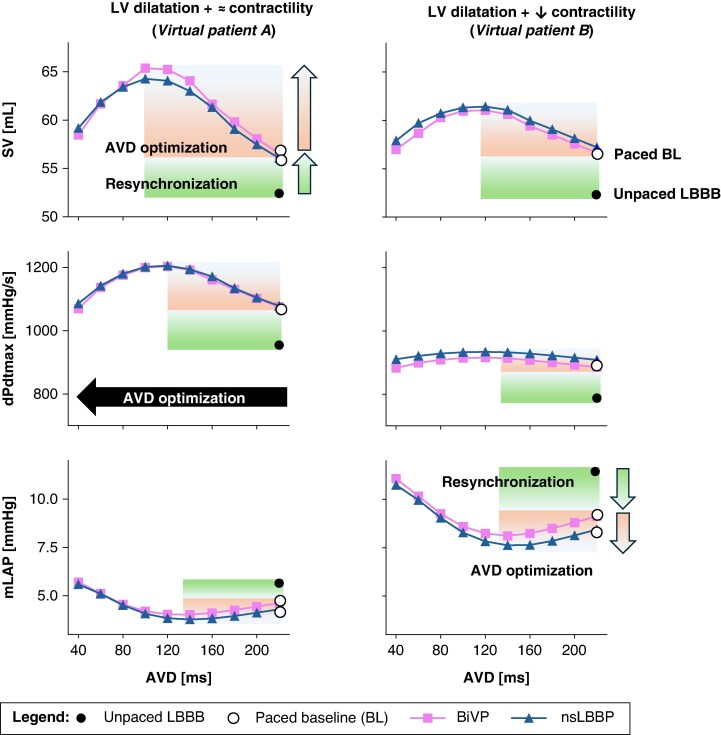
Effect of shortening AVD on SV and dPdtmax, representing the acute response, and mLAP representing the chronic response, starting from LBBB baseline at an AVD of 220 ms and applying BiVP (pink squared line) and nsLBBP (blue triangle line). The left panel shows the results in the presence of LBBB with dilatation and mantained contractility, while the right panel presents the results in the presence of LBBB with dilatation and decreased contractility. The green arrow and green square indicate the increase (for SV and dPdtmax) and decrease (for mLAP) due to pacing, while the orange arrow and orange square represent the increase (for SV and dPdtmax) and decrease (for mLAP) due to AVD optimization. AVD, atrio-ventricular delay; BiVP, biventricular pacing; dPdtmax, maximum rate of pressure change; LBBB, left bundle branch block; LV, left ventricle; mLAP, mean left atrial pressure; nsLBBP, non-selective left bundle branch pacing; SV, stroke volume.

**Figure 4:**
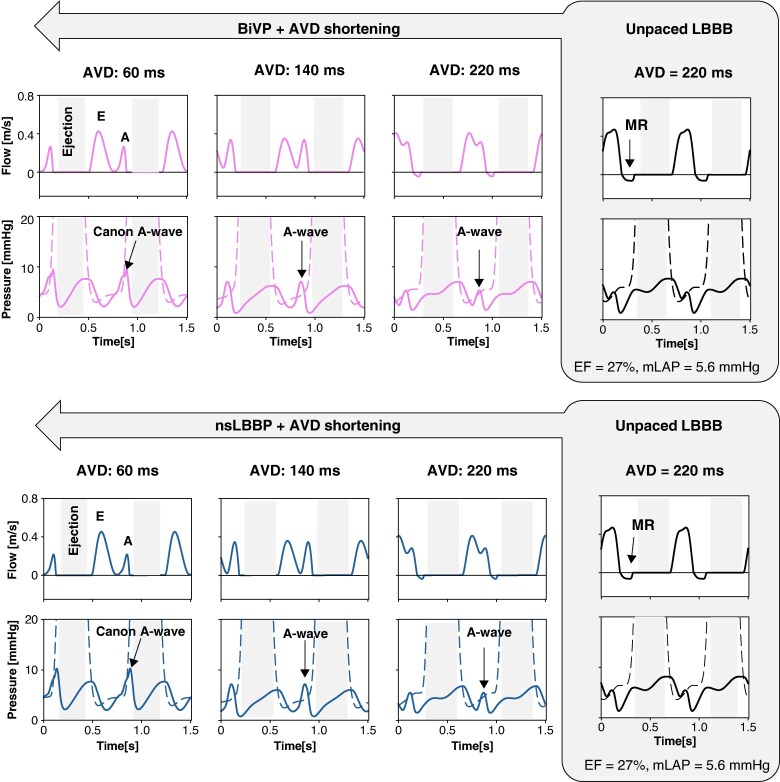
Effect of shortening AVD on pressure and flow signals in the presence of LBBB with LV dilatation and maintained contractility (*virtual patient A*). The upper panel illustrates AVD optimization during BiVP , while the lower and right panels show results from AVD optimization during nsLBBP (blue lines). In both panels, mitral flow velocity and pressures are presented, with LA pressure shown by the solid line and LV pressure shown by the dotted line. These measurements are shown from the unpaced LBBB baseline at an AVD of 220 ms to pacing with a short AVD of 60 ms. The figure also presents mitral flow velocity and pressures before and after pacing at long (220 ms), medium (140 ms), and short (60 ms) AVDs. At long AVD, mitral flow is characterized by MR, while at short AVD LA pressure is characterized by a prominent canon A-wave. AVD, atrio-ventricular delay; BiVP, biventricular pacing; CRT, cardiac resynchronization therapy; EF, ejection fraction; LA, left atrium; LBBB, left bundle branch block; LV, left ventricle; mLAP, mean left atrial pressure; MR, diastolic mitral regurgitation; SV, stroke volume.

*Figure 3* illustrates the effects of varying AVD with BiVP (pink square-dashed line) and nsLBBP (blue triangle-dashed line) on SV, dPdtmax, and mLAP for virtual patients A and B, with maintained (left column) and reduced (right column) contractility, respectively. The optimization curves for BiVP were comparable to those for nsLBBP. These plots serve as examples to highlight the contributions of both resynchronization (*Figure 4*: green arrow) and AVD optimization (*Figure 4*: orange arrow). Although the haemodynamic improvement as a result from resynchronization was comparable between phenotypes, the haemodynamic improvement attributable to AVD shortening exhibited phenotype-specific variation. For analytical clarity, we focus on BiVP outcomes. In the virtual patient model with maintained contractility, AVD shortening yielded an absolute SV increase of 8.8 mL at the optimal AVD of 100 ms during BiVP, representing a 16% improvement relative to the paced baseline at AVD = 220 ms. Conversely, the contractile dysfunction model demonstrated a more modest haemodynamic response, with an absolute SV increment of 4.3 mL at an optimal AVD of 120 ms, constituting an 8% increase from the paced baseline condition at AVD = 220 ms. These differential percentage improvements in SV are consistent with findings reported in previous investigations.^28^

The maintained contractility model showed significant dPdtmax improvement (+128 mmHg/s at AVD = 120 ms, 12% increase), while the contractile dysfunction model showed minimal increase (+30 mmHg/s, 3%). Atrio-ventricular delay shortening reduced mLAP by 0.8 and 1.0 mmHg in maintained and reduced contractility models, respectively.

*Figure 4* shows the effect of AVD shortening on pressure and flow signals during BiVP (upper panel) and nsLBBP (lower panel) in *virtual patient A*. At baseline (unpaced LBBB), mitral flow exhibited fused E/A-waves and diastolic mitral regurgitation (MR). Shortening AVD from 220 to 140 ms separated these waves and reduced MR. At AVD ≤ 60 ms, A-wave truncation occurred with increased mLAP due to atrial contraction against closed mitral valve, creating canon A-waves in the left atrium (LA) pressure signal.

**Figure 5:**
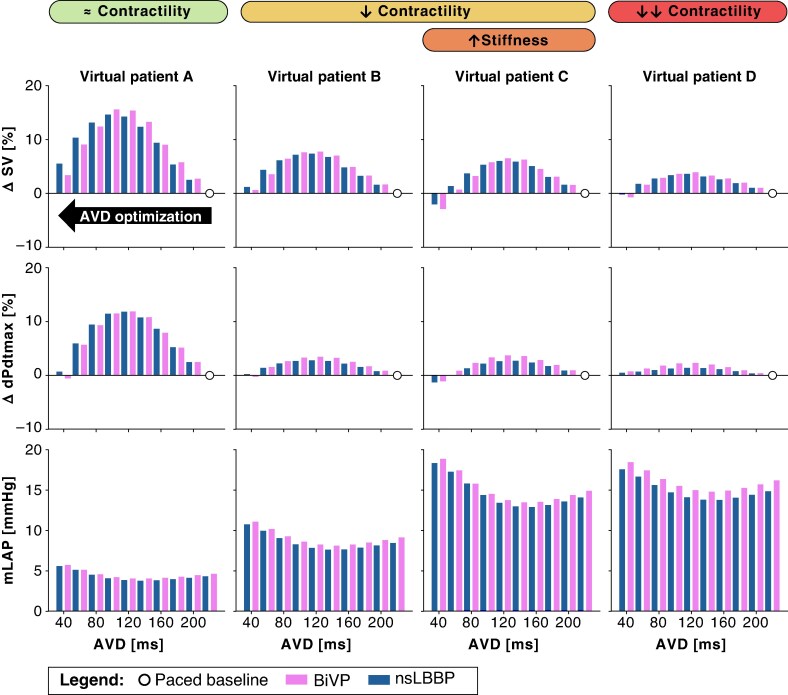
Haemodynamic effects of improving AV coupling during BiVP pacing (pink) and nsLBBP (blue). The figure shows the relative changes in haemodynamic function by shortening AVD, compared to baseline values for BiVP and nsLBBP at 220 ms (white dot). Virtual patient A shows LBBB with dilatation and preserved contractility (green); Virtual patient B represents LBBB with dilatation and reduced contractility (yellow); Virtual patient C demonstrates LBBB with dilatation, reduced contractility, and severe increase in passive stiffness (yellow + orange); Virtual patient D shows LBBB with severe reduction in contractility (red). The optimal AVD corresponds to the maximum SV, maximum dPdtmax, and minimum mLAP. AVD, atrio-ventricular delay; BiVP, biventricular pacing; mLAP, mean left atrial pressure; LBBB, left bundle branch block; nsLBBP, non-selective LBB pacing; SV, stroke volume.

*Figure 5* and *Table 4* summarize haemodynamic effects across all phenotypes. Lowest mLAP was achieved with an AVD that was 20 ms longer than the AVD associated with maximal SV and dPdtmax (*Table 4*). *Figure 5* illustrates the percentage change relative to baseline for SV and dPdtmax, as well as the absolute change in mLAP, following pacing at an AVD of 220 ms (indicated by the white dot as the paced baseline). Atrio-ventricular delay shortening yielded greater SV and dPdtmax improvements in maintained contractility models (BiVP: SV +15%, dPdtmax +12%) vs. reduced contractility (SV +8%, dPdtmax +3%). All phenotypes showed comparable mLAP decreases (∼1 mmHg).

**Table 4:** Simulated haemodynamic data of optimal AVD and paced baseline values for BiVP and nsLBBP

	BiVP		nsLBBP	
	Paced baseline	AVDopt	Paced baseline	AVDopt
Virtual patient A				
AVD (ms)	220	100	220	100
SV (mL)	56.5	65.3	56.05	64.2
dPdtmax (mmHg/s)	1075.4	1200.0	1078.0	1205.3
mLAP (mmHg)	4.61	4.03 (130)*	4.2	3.8 (140)*
Virtual patient B				
AVD (ms)	220	120	220	120
SV (mL)	56.6	61.03	57.2	61.4
dPdtmax (mmHg/s)	885.2	915.7	908.4	933.8
mLAP (mmHg)	9.12	8.2 (140)*	8.4	7.6 (140)*
Virtual patient C				
AVD (ms)	220	120	220	120
SV (mL)	54.7	58.3	55.1	58.43
dPdtmax (mmHg/s)	873.1	905.35	886.1	910.0
mLAP (mmHg)	14.9	13.73 (140)*	13.8	12.7 (140)*
Virtual patient D				
AVD (ms)	220	120	220	
SV (mL)	55.1	57.29	55.9	57.9
dPdtmax (mmHg/s)	733.1	749.7	742.0	752.3
mLAP (mmHg)	16.2	14.9 (140)*	14.8	13.8 (140)*

The values in ()* represent the AVDopt for mLAP which is found to be different from the one of SV and dPdtmax.

AVD, atrio-ventricular delay; AVDopt, optimal atrio-ventricular delay; dPdtmax, maximum rate of LV pressure change; LBBB, left bundle branch block; LV, left ventricular; mLAP, mean left atrial pressure; nsLBBP, non-selective LBB pacing; SV, stroke volume.

### Effect of atrio-ventricular delay variation on exercise haemodynamics

**Figure 6:**
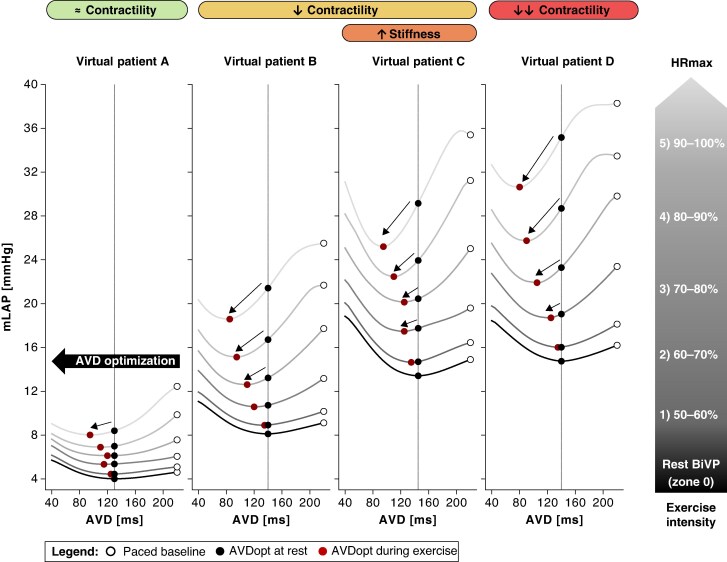
Simulation-based assessment of the relative importance of AV delay optimization BiVP, across six exercise intensity zones: zone 0 (rest), zone 1 (50–60% HRmax), zone 2 (60–70% HRmax), zone 3 (70–80% HRmax), zone 4 (80–90% HRmax), and zone 5 (90–100% HRmax). The white dot marks the value of mLAP at an AVD of 220 ms during paced baseline. The vertical black line represents the optimal AVD at rest, while the black dot indicates the corresponding mLAP at this resting optimum. The red dot shows the mLAP at the optimal AVD for each level of exercise intensity. Starting from the resting condition with BiVP (depicted by the black parabolic line), the optimal AVD shifts to shorter values across all six substrates during exercise, as indicated by the black arrow. AVD, atrio-ventricular delay; BiVP, biventricular pacing; CO, cardiac output; HR, heart rate; HRmax, maximum heart rate; mLAP, mean left atrial pressure; Opt, optimal.

*Figure 6* shows the effects of BiVP at different AVD settings on filling pressure at the increasing levels of exercise intensity across phenotypes. The nsLBBP results are provided as Supplementary materials (Supplementary material online, *Figure S2* and Supplementary material online, *Table S1*). Black dots represent rest-optimized AVD mLAP values, white dots show baseline mLAP (AVD = 220 ms), and red dots indicate optimal AVD values per exercise zone. Parabolic curve steepness increased during exercise, particularly at longer AVDs, indicating enhanced sensitivity of filling pressure to AVD at higher heart rates. Overall, optimal AVDs became shorter with increasing exercise levels in all virtual patients.

**Figure 7:**
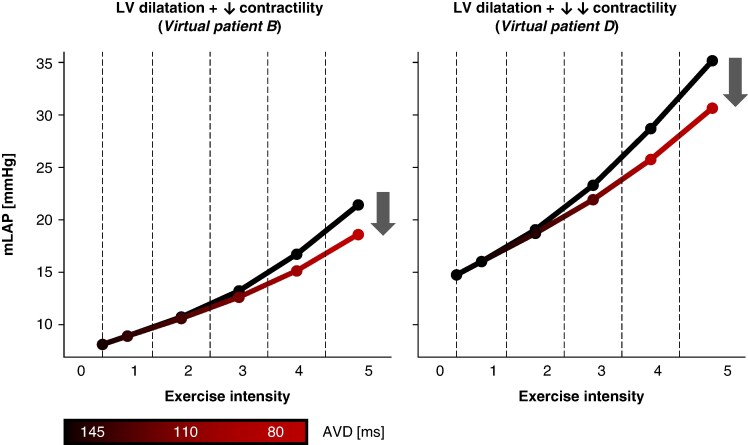
Mean left atrial pressure (mLAP) as a function of exercise intensity and the considered optimal AVD. The black line represents the mLAP values when the optimal AVD identified at baseline is maintained across all exercise stages. The gradient black-to-red line illustrates the mLAP values when the AVD is optimized for each individual exercise stage. The definition of the exercise intensity zones can be found in the caption of *Figure 6*. AVD, atrio-ventricular delay; LV, left ventricle.

Notable differences emerged between phenotypes (*Table 5*): patients with reduced contractility and increased stiffness (*Figure 6*: virtual patients B–D) showed significant filling pressure decreases with exercise-specific AVD shortening compared to maintaining rest-optimized AVD. Patients with preserved contractility (*Figure 6*: virtual patient A) showed minimal pressure reduction. To emphasize the relative importance of AVD optimization during exercise compared to rest, *Figure 7* demonstrates that fixed rest-optimized AVD during exercise increased mLAP, while exercise-specific AVD adjustment reduced it, particularly in patients with elevated baseline filling pressures.

**Table 5:** Simulated mLAP values during BiVP for each virtual patient across different exercise zones, including the resting condition.

	Exercise intensity	0	1	2	3	4	5
Virtual patient A	AVDopt	130	130	130	130	130	130
	Rest (ms)						
	mLAP (mmHg)	4.0	4.4	5.36	6.1	6.9	8.3
	AVDopt Exercise (ms)	130	125	115	120	110	95
	mLAP (mmHg)	4.0	4.4	5.3	6.1	6.8	8.0
Virtual patient B	AVDopt	140	140	140	140	140	140
	Rest (ms)						
	mLAP (mmHg)	8.1	8.9	10.7	13.2	16.7	21.4
	AVDopt Exercise (ms)	140	135	120	110	95	85
	mLAP (mmHg)	8.1	8.9	10.6	12.6	15.1	18.6
Virtual patient C	AVDopt	145	145	145	145	145	145
	Rest (ms)						
	mLAP (mmHg)	13.4	14.7	17.7	20.4	23.9	29.1
	AVDopt Exercise (ms)	145	135	125	125	110	95
	mLAP (mmHg)	13.4	14.6	17.4	20.1	22.4	25.1
Virtual patient D	AVDopt	145	145	145	145	145	145
	Rest (ms)						
	mLAP	14.7	116.0	19.0	23.3	28.7	35.2
	AVDopt Exercise (ms)	140	135	125	105	90	85
	mLAP	14.7	16.0	18.7	21.9	25.7	30.6

The table presents mLAP values for both the optimal AVD at rest and the AVD optimized for each respective exercise zone.

AVD, atrio-ventricular delay; AVDopt, optimal atrio-ventricular delay; mLAP, mean left atrial pressure.

## Discussion

To the best of our knowledge, this is the first *in silico* study investigating the effect of different HF phenotypes on the acute and chronic haemodynamic responses to BiVP and conduction system pacing with varying AVD at rest and during exercise conditions.

### Impact of pacing modalities on atrio-ventricular delay optimization

While BiVP has been the gold standard pacing strategy in CRT candidates for many years, nsLBBP has emerged as a promising alternative due to its potential for more precise resynchronization by directly targeting the left bundle branch. By pacing the LV more effectively, nsLBBP may offer superior benefits in terms of filling pressures and overall cardiac function. Our simulations of nsLBBP indeed showed more favourable improvements in filling pressure compared to BiVP. This enhanced effectiveness of nsLBBP may be attributed to its ability to better synchronize the left ventricle, resulting in more efficient pump function.^18^ These findings align with the literature^13^ suggesting that conduction system pacing as a novel pacing strategy, may offer advantages over traditional BiVP in patients with specific conduction abnormalities, potentially leading to better clinical outcomes.

### Dependence on myocardial phenotype

Previous studies^2,34^ focused on the benefit of adjusting AVD in CRT cohorts without analysing the potential confounding effects of underlying myocardial disease. Our computer simulations suggest that virtual patients with LV contractile dysfunction and increased myocardial stiffness may not benefit as much from cardiac pacing; resynchronization alone may not be enough for cases with stiff myocardium. A more effective approach to improve outcomes could be to adjust the AVD to allow for more time for ventricular filling. This is particularly crucial during exercise, as explained further in the next section, where patients with LV contractile dysfunction and increased myocardial stiffness are more responsive to AVD changes compared to those with normal contractility and stiffness.

### Rest vs. exercise

Exercise represents the state in which most patients with HF become symptomatic. The importance of AVD optimization during exercise has already been assessed and demonstrated by different studies,^35–37^ and the inclusion of rate-adaptive algorithms have been introduced in CRT devices.^13,30,31,38–40^ However, these studies also showed that the optimal AVD at increased HR differed between patients and could be higher or lower than the one at rest. Additionally, the analysis of haemodynamics primarily focused on SBP due to its easier acquisition. It thereby overlooked the potentially stronger limiting factor for the cardiovascular system’s exercise capacity, which is filling pressure. Most of these studies conclude that the choice of AVD is patient-specific without addressing which subgroup of patients would benefit the most from AVD optimization.^37^

Thanks to the model, it was possible to simulate different degrees of exercise and analyse cardiac pump function in terms of CO and filling pressure, which is challenging to measure in the clinic, among the different virtual patient phenotypes. The results demonstrated that shortening the paced AVD improved exercise haemodynamics by reducing filling pressure while simultaneously increasing CO and HR.

The most significant improvement in exercise haemodynamics, in terms of filling pressure, were seen in virtual patients with increased operating chamber stiffness. However, these patients did not experience a significant improvement in SV at rest, implying that acute haemodynamic response to AVD optimization in terms of SV and SBP did not accurately reflect potential benefits during exercise.

### Impact on filling pressure

We observed a consistent decrease in filling pressure with shortening of the AVD for all the six substrates, reaching a minimum value that corresponds to the potentially optimal AVD. The model demonstrated notable changes in LAP (left atrial pressure) waveform when varying the AVD (*Figure 4*). These observations are consistent with the findings of Chan *et al.*^41^ highlighting how left atrial pressure and its waveform can be affected by AVD optimization. Prolonged AVD resulted in functional MR, causing a more rapid pressure rise during early atrial filling. Conversely, a short AVD led to a prominent canon A-wave in the LA pressure signal because of the premature closure of the mitral valve, while the LA was still contracting and building up pressure. Both conditions are particularly problematic for patients with high mLAP, in whom a suboptimal AVD further increases mLAP and, hence, the chance for pulmonary oedema.

### Instantaneous vs. chronic haemodynamic response

In clinical settings, measuring SV and dPdtmax using echo-Doppler requires highly skilled experts and a considerable amount of time for data acquisition. As an alternative, SBP or pulse pressure is often utilized to analyse the effects of AVD optimization.^3,28,42^ An increase in SV, indicating an improvement in systolic performance, is typically accompanied by a rise in SBP.

However, the immediate increment in SBP observed during the first beats after changing the AVD may decay in subsequent beats due to the vascular peripheral response. Our model was able to reproduce these results when considering a healthy myocardium, however, in cases of hypocontractility and increased passive stiffness, the phenomenon of secondary decline in pressure was less pronounced.

## Clinical implications

Optimizing AVD settings CRT can improve treatment outcomes,^1,13,30,43^ though its clinical value remains debated. Scepticism about performing AVD optimization in daily clinical practice stems from the lack of a gold standard on AV optimization techniques and guidelines for selecting the correct patients.^11^ Consequently, clinicians are forced to rely on nominal (‘out-of-the-box’) settings and a ‘one-size-fits-all’ approach. Our virtual patient study suggests that a more personalized, phenotype-specific, and rate-adaptive optimization procedure is needed to deliver optimal pacing therapy at rest as well as during physical activity. More specifically, our simulations suggest that patients with increased operating stiffness and, as a result, higher filling pressure at baseline would benefit the most from AVD optimization. Furthermore, our findings emphasize the need to consider symptom worsening during exertion, which may occur due to an unnecessary increase in filling pressure resulting from suboptimal AVD settings.

Incorporating AVD optimization into therapeutic strategies may offer a valuable approach to enhance treatment efficacy in both HFrEF and heart failure with preserved ejection fraction (HFpEF) populations. This potential broader application is supported by van Loon *et al.*^44^ who demonstrated in a combined clinical-computational study that moderately accelerated atrial pacing can acutely reduce mLAP in patients with HFpEF. However, their investigation revealed that higher pacing rates induce decremental atrio-ventricular conduction, wherein electrical impulse propagation through the AV node progressively slows with increased pacing frequency. This physiological adaptation results in PR interval prolongation, consequently elevating mLAP. These findings suggest that strategic AVD optimization through targeted regulation of atrio-ventricular timing could potentially benefit HFpEF patients by improving haemodynamic parameters, complementing the benefits observed in HFrEF patients in our current study.

## Study limitations

In this virtual patient study, we did not allow for conduction through the AV-node, even though in real patients, there could be preserved conduction. As explained in the methods, we made this decision to focus solely on the impact of changes in AV dynamics, without any influence from possible fusion pacing. We also applied the same relationship to all virtual patients between CO–HR when simulating exercise, although this relationship may vary from patient to patient. Despite the high inter-subject variability, we believe that this relation might well capture the overall behaviour of HFrEF patients in the absence of chronotropic incompetence.

All computer simulations resulted in acute (i.e. change in SV and dPdtmax) and chronic haemodynamic response (i.e. change in mLAP) to resynchronization and AVD optimization but did not take into account the long-term structural remodelling effects. Nevertheless, we believe that long-term benefits of decreased filling pressure and improved symptoms would positively impact the patient’s condition. Finally, the presented study should be considered a hypothesis-generating study where only virtual patient simulations were used in the absence of real clinical data. Even though clinical data are needed to study the complexity of AVD optimization and to determine its effect on patient outcome, we believe that the mechanistic knowledge obtained from our virtual patient study will help to optimize the design and effectiveness of device algorithms and related real-world clinical studies.

## Conclusions

Our virtual patient simulations revealed that the haemodynamic impact of AVD optimization during cardiac resynchronization is strongly dependent on the underlying myocardial phenotype and that this dependence is even more pronounced during exercise. Left ventricular filling pressure is most sensitive to AVD in hearts with increased LV chamber stiffness, in particular during exercise. The rate at which the optimal AVD decreases with each beat per minute increase in HR is steeper in hearts with increased LV chamber stiffness, as compared to hearts with preserved LV compliance. Furthermore, our results suggest that acute haemodynamic response (in terms of dPdtmax and SV) to AVD optimization at rest is not necessarily representative of the potential haemodynamic benefit during exercise.

## Supplementary Material

euaf082_Supplementary_Data

## Data Availability

The data underlying this article will be shared on reasonable request to the corresponding author.
