# Supplementary material for: Impact of myocardial phenotype on optimal atrioventricular delay settings during biventricular and left bundle branch pacing at rest and during exercise: insights from a virtual patient study
Source: Europace. 2025 Apr 8;27(4):euaf082. doi: 10.1093/europace/euaf082 (PMC12035189; doi:10.1093/europace/euaf082)
Supplement: euaf082_Supplementary_Data [file euaf082_supplementary_data.docx]

**Supplementary Materials**

***Impact of myocardial phenotype on optimal atrioventricular delay settings during biventricular and left bundle-branch pacing at rest and during exercise: insights from a virtual patient study***

**
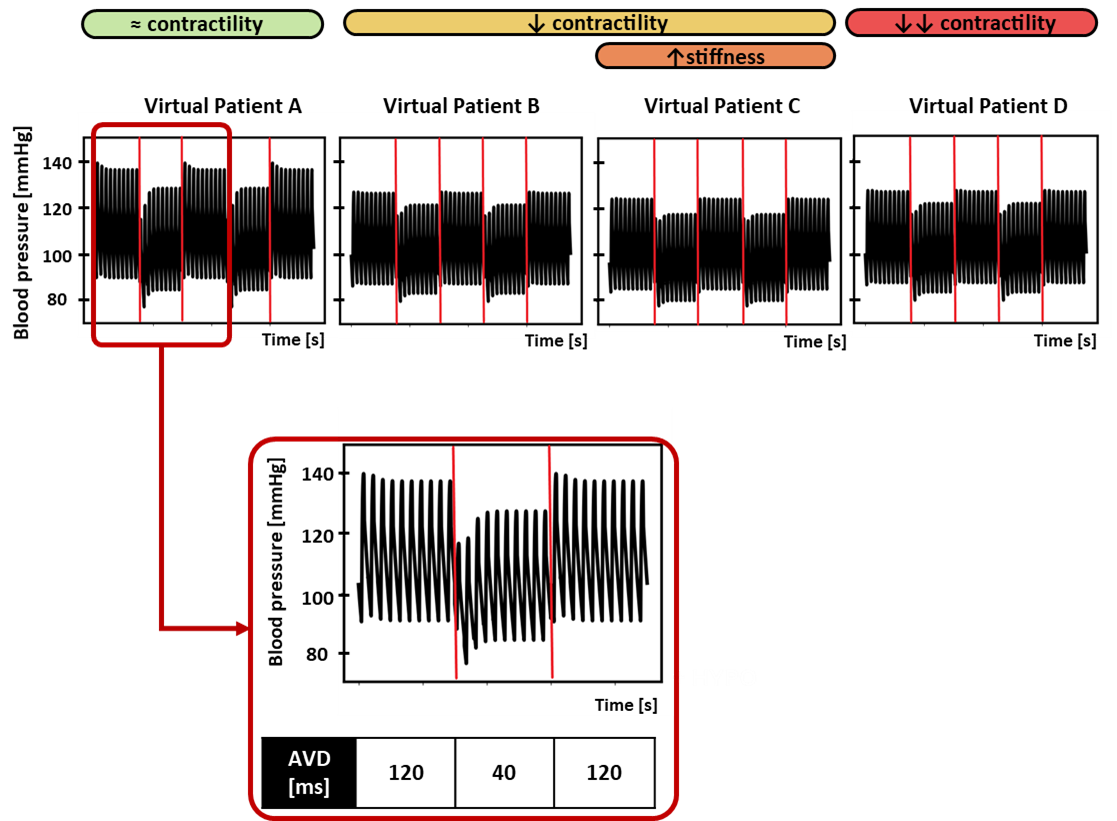
**

**Figure S1:** Beat-to-beat analysis of simulated systolic blood pressure (SBP) during alternation of AVD setting. The red box shows an example of a prompt change in blood pressure when the AVD is changed. AVD, atrioventricular delayLBBB, left bundle branch block; SBP, systolic blood pressure; Stiff; stiffness.

**
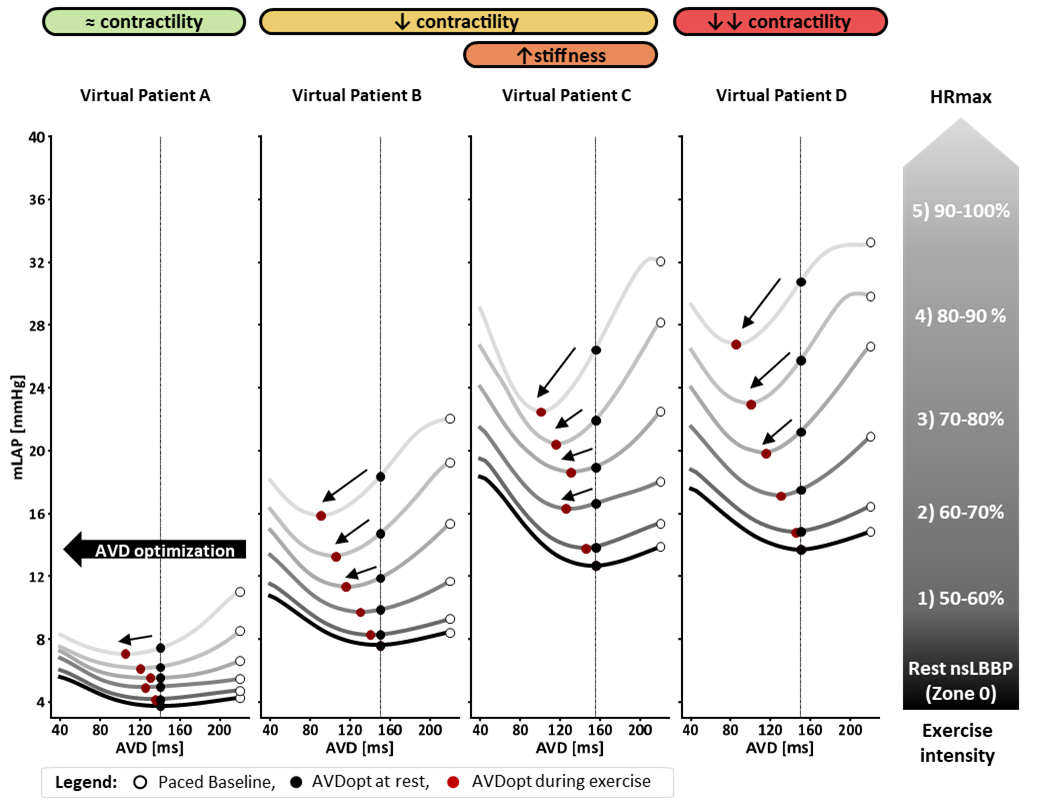
**

**Figure S2:** AVD optimization during exercise after nsLBBP pacing

**Table S1:** Simulated mLAP values during nsLBBP for each virtual patient across different exercise zones, including the resting condition. The table presents mLAP values for both the optimal AVD at rest and the AVD optimized for each respective exercise zone. AVDopt, optimal atrioventricular delay; mLAP, mean left atrial pressure.

|  | **Exercise intesity** | **0** | **1** | **2** | **3** | **4** | **5** |
| --- | --- | --- | --- | --- | --- | --- | --- |
| **Virtual patient A** | AVDopt  Rest [ms] | 140 | 140 | 140 | 140 | 140 | 140 |
|  | mLAP [mmHg] | 3.7 | 4.1 | 4.9 | 5.5 | 6.2 | 7.4 |
|  | AVDopt Exercise [ms] | 140 | 135 | 125 | 130 | 120 | 105 |
|  | mLAP [mmHg] | 3.7 | 4.1 | 4.9 | 5.5 | 6.1 | 7.0 |
|  | | | | | | | |
| **Virtual Patient B** | AVDopt  Rest [ms] | 150 | 150 | 150 | 150 | 150 | 150 |
|  | mLAP [mmHg] | 7.6 | 8.3 | 9.8 | 11.9 | 14.7 | 18.3 |
|  | AVDopt Exercise [ms] | 150 | 140 | 130 | 115 | 105 | 90 |
|  | mLAP [mmHg] | 7.6 | 8.3 | 9.7 | 11.3 | 13.27 | 15.87 |
|  | | | | | | | |
| **Virtual Patient C** | AVDopt  Rest [ms] | 155 | 155 | 155 | 155 | 155 | 155 |
|  | mLAP [mmHg] | 12.6 | 13.8 | 16.6 | 18.9 | 21.9 | 26.4 |
|  | AVDopt Exercise [ms] | 155 | 145 | 125 | 130 | 115 | 100 |
|  | mLAP [mmHg] | 12.6 | 13.7 | 16.3 | 18.6 | 20.4 | 22.47 |
|  | | | | | | | |
| **Virtual Patient D** | AVDopt  Rest [ms] | 150 | 150 | 150 | 150 | 150 | 150 |
|  | mLAP | 14.7 | 116.0 | 19.0 | 23.3 | 28.7 | 35.2 |
|  | AVDopt Exercise [ms] | 140 | 135 | 125 | 105 | 90 | 85 |
|  | mLAP | 13.6 | 14.8 | 17.5 | 21.2 | 25.7 | 30.7 |
